# Genome Sequences of *Streptococcus thermophilus* Strains MTH17CL396 and M17PTZA496 from Fontina, an Italian PDO Cheese

**DOI:** 10.1128/genomeA.00067-14

**Published:** 2014-02-13

**Authors:** Laura Treu, Veronica Vendramin, Barbara Bovo, Stefano Campanaro, Viviana Corich, Alessio Giacomini

**Affiliations:** a Department of Environmental Engineering, Technical University of Denmark, Kongens Lyngby, Denmark; b Department of Agronomy, Food, Natural Resources, Animals and Environment (DAFNAE), University of Padova, Padua, Italy; c Department of Biology, University of Padova, Padua, Italy

## Abstract

Here is presented the whole-genome sequences of *Streptococcus thermophilus* strains MTH17CL396 and M17PTZA496, isolated from fontina protected designation of origin (PDO) cheese in the Valle d’Aosta Region (Italy). *S. thermophilus* is a lactic acid bacterium widely present in dairy products, and these are the first publicly available genome sequences of *S. thermophilus* strains isolated from cheese.

## GENOME ANNOUNCEMENT

The economic value of fermented foods is relevant worldwide, and dairy production alone accounts for an annual economic value of $54.2 billion, whereas the cheese market is even larger ($74.4 billion) (1). To explore their potential functional properties in detail, we sequenced two *Streptococcus thermophilus* strains, MTH17CL396 and M17PTZA496, originally isolated from fontina cheese (a semihard cheese from raw cow milk) in Italy. Both strains showed high biofilm production activities; strain M17PTZA496 is capable of utilizing galactose, contrary to MTH17CL396.

The sequencing of *S. thermophilus* MTH17CL396 and M17PTZA496 was performed at the Ramaciotti Centre, Sydney, Australia. Respectively, 407-fold and 107-fold sequencing coverages were obtained using an Illumina MiSeq platform with 1-kb mate-pair libraries and paired-end reads (2 × 250 bp). The files generated were assembled with Velvet version 1.2.10 (2) and ABySS software version 1.3.5 (3), with an optimal k-mer size of 131, and the consensus sequences of the two assemblies were manually compared. For the MTH17CL396 strain, 49 scaffolds with a total size of 1,822,425 bp, with an overall G+C content of 38.9%, were obtained; strain M17PTZA496 gave 72 scaffolds and 2,064,069 bp of total genome size, with an overall G+C content of 38.8%. Both strains had the scaffolds assembled into a single circular chromosome, aligning the scaffolds against the reference genome of *S. thermophilus* CNRZ1066 (assembly no. ASM1184v1). No plasmid sequences were detected by BLAST analysis in MTH17CL396, while some matches were found for M17PTZA496 (scaffolds 58, 63, and 64). Furthermore, several similarities with *Streptococcus macedonicus* ACA-DC198 DNA (assembly no. ASM28363v1) were found for strain M17PTZA496.

Protein-coding open reading frames (ORFs) were predicted and annotated using the RAST annotation server (4). The MTH17CL396 genome is predicted to contain 1,935 protein-coding genes and 56 RNA genes, while the M17PTZA496 genome has 2,221 ORFs and 89 RNA genes. The *S. thermophilus* MTH17CL396 genome does not contain prophage sequences or transposase-coding genes, while 25 clusters of regularly interspaced short palindromic repeats (CRISPRs) or CRISPR-associated clusters were found. In contrast, 22 phage-associated sequences, no genes related to transposases, and only 4 CRISPRs were detected in M17PTZA496.

A comparison with the reference genome of *S. thermophilus* CNRZ1066 highlighted 233 exclusive predicted coding sequences (CDSs) for MTH17CL396 and 359 CDSs for M17PTZA496. The metabolic network has been reconstructed, using the RAST server, for both strains, and sequences of particular interest were found. In particular, strain M17PTZA496 includes an interesting number of “extracellular polysaccharide biosynthesis of streptococci” and “exopolysaccharide biosynthesis” subsystem features. Specific genes were assigned to several pathways for carbohydrate and amino acid metabolism. Specific free amino acid patterns characterize different cheese varieties, and therefore, this ability to biosynthesize amino acids might improve cheese quality during ripening (5).

These data are intended to increase the availability of the genomes of *S. thermophilus* strains of dairy origin in order to better understand their biodiversity and their known and potential technological properties.

### Nucleotide sequence accession numbers.

These whole-genome shotgun projects have been deposited at DDBJ/EMBL/GenBank under the accession no. AZJS00000000 for *S. thermophilus* strain MTH17CL396 and AZJT00000000 for strain M17PTZA496. The versions described in this paper are, respectively, AZJS01000000 and AZJT01000000.
